# Probabilistic linkage of multiple data sources for estimating prevalence of problem drug users in England in 2018/19.

**DOI:** 10.23889/ijpds.v7i3.1904

**Published:** 2022-08-25

**Authors:** Abdelmajid Djennad, Stefan Jahr, Ross Harris, Andre Charlett

**Affiliations:** 1 Office for National Statistics; 2 Department of Health and Social Care

## Objectives

Problem drug use (PDU) prevalence is an essential part of evidence base to formulate policy, inform service provision, and assess interventions. Opiate and crack use, particularly injecting, is associated with infectious diseases. Multiple data sources, where PDU are observed, are linked to estimate the hidden population of PDU using capture-recapture.

## Approach

Datasets consisted of (1) community treatment, (2) prison treatment, (3) probation (Ministry of Justice), and (4) drug-related deaths, for financial year 2018/19. The following offenders’ attributes and geography are used: first and second initials, date of birth, gender, multiple areas of residence and area of prison release. Geography information consists of local authority code and region name. Probabilistic linkage approach of Fellegi–Sunter is applied through FastLink package in R, where pairs of records are classified as match, possible match, or non-match at 85% threshold. Estimates of error rates, sensitivity and specificity are used to test the results.

## Results

There were 138,341 records in community treatment, 41,700 records in prison, 18,849 in probation and 2,368 deaths from opiate or cocaine. The total number of individuals observed in at least one linked dataset is estimated at 170,307 records at 85% threshold. The number of exact and possible matches overlapping between community treatment and prison is estimated at 17,544 records, from which 13,731 (78.3%) are exact matches, and 3,813 (21.7%) are possible matches at 85% threshold. The number of exact and possible matches overlapping between linked community treatment/prison and probation is estimated at 12,586 records, from which 9,872 (78.4%) are exact and 2,714 (21.6%) are possible matches at 85% threshold. False discovery rate was estimated at 0.3%, sensitivity and specificity were 99.8%.

## Conclusion

Information on PDUs attending community treatment is poor and relying on exact matches underestimate the overlap between data sources. This tend to bias hidden population estimates derived from capture-recapture. Probabilistic approaches, and use of multiple candidate geographic areas for matching, maximise linkages between individuals and thus likely to improve estimates.

